# Human infrapatellar fat pad mesenchymal stem cells show immunomodulatory exosomal signatures

**DOI:** 10.1038/s41598-022-07569-7

**Published:** 2022-03-04

**Authors:** Dimitrios Kouroupis, Lee D. Kaplan, Thomas M. Best

**Affiliations:** 1 Department of Orthopedics, UHealth Sports Medicine Institute, University of Miami, Miller School of Medicine, Miami, FL USA; 2 Diabetes Research Institute & Cell Transplantation Center, University of Miami, Miller School of Medicine, Miami, FL USA; 3 Department of Orthopaedics, Division of Sports Medicine, Diabetes Research Institute, Cell Transplant Center, University of Miami, Miller School of Medicine, 1450NW 10th Ave, Room 3014, Miami, FL 33136 USA

**Keywords:** Stem-cell therapies, Mesenchymal stem cells

## Abstract

Within the human knee infrapatellar fat pad (IFP) and synovium, resident synoviocytes and macrophages contribute to the onset and progression of inflammatory joint diseases. Our hypothesis is that IFP-derived mesenchymal stem cells (IFP-MSC) robust immunomodulatory therapeutic effects are largely exerted via their exosomal (IFP-MSC EXOs) secretome by attenuating synoviocytes and macrophages pro-inflammatory activation. IFP-MSC EXOs showed distinct miRNA and protein immunomodulatory profiles. Reactome analysis of 24 miRNAs highly present in exosomes showed their involvement in the regulation of six gene groups, including immune system. Exosomes were enriched for immunomodulatory and reparative proteins that are involved in positive regulation of cell proliferation, response to stimulus, signal transduction, signal receptor activity, and protein phosphorylation. Stimulated synoviocytes or macrophages exposed to IFP-MSC EXOs demonstrated significantly reduced proliferation, altered inflammation-related molecular profiles, and reduced secretion of pro-inflammatory molecules compared to stimulated alone. In an acute synovial/IFP inflammation rat model, IFP-MSC EXOs therapeutic treatment resulted in robust macrophage polarization towards an anti-inflammatory therapeutic M2 phenotype within the synovium/IFP tissues. Based on these findings, we propose a viable cell-free alternative to MSC-based therapeutics as an alternative approach to treating synovitis and IFP fibrosis.

## Introduction

The synovium and IFP combine as a single anatomic and functional unit^1^ and serve as a site of immune cell infiltration and origin of pro-inflammatory and articular cartilage (AC) degradative molecules. In pathological conditions, deranged chondrocyte metabolism results in release within the joint of AC degradation products and other damage-associated molecular patterns (DAMPs)^2–5^ that incite local responses within the synovium/IFP tissues. These responses include: (1) local infiltration of immune cells such as macrophages, (2) shift of resident macrophages to a pro-inflammatory M1 phenotype^2,6^, (3) production of AC-degrading proteases (*e.g.,* MMPs), and pro-inflammatory cytokines, adipokines and other mediators, and (4) sustained synovitis and progressive IFP fibrosis.

Mesenchymal Stem Cell (MSC)-based therapy has gained attention as a potential therapeutic alternative for such inflammatory conditions, given their immunomodulatory and trophic effects involving anti-inflammatory and anti-fibrotic actions^7^. IFP tissue constitutes a promising alternative source of MSC to other adult/foetal tissues such as the “standard" bone marrow (BM), given its anatomical relationship with intra-articular structures and its pivotal role in joint homeostasis and disease^8^. Based on this, we recently reported human IFP derived Mesenchymal Stem Cells (IFP-MSC) acquiring a potent immunomodulatory phenotype, and actively reversing inflammation and fibrosis linked with macrophage polarization from an M1 in disease to an M2 phenotype after engraftment into areas of active synovitis/IFP fibrosis^9–14^.

Exosomes are nanosized (50–200 nm) extracellular vesicles (EVs) generated via the endosomal pathway^15^, and secreted by numerous cells in response to their surrounding milieu. Thus, their contents (i.e., cargo) and lipid shell may carry information that reflects particular changes in the parental cells’ microenvironment specifying intrinsic communications to proximal or distal sites. Previous studies have isolated and characterized exosomes from various MSC sources (i.e. bone marrow, umbilical cord, adipose tissues), confirming their strong anti-inflammatory, anti-fibrotic and angiogenesis-remodeling capacities^16^. Herein, our hypothesis is that IFP-MSC robust immunomodulatory effects are largely exerted via their exosomal secretome by attenuating synoviocytes and macrophages proliferation and pro-inflammatory activation. Our overarching goal is to determine whether the resulting IFP-MSC derived exosomes (IFP-MSC EXOs) are able to recapitulate our reported therapeutic activity with IFP-MSC^9–13^. Cell-free products have advantages compared with their replicative cell-based counterparts, including reduced variability, standardization, easy storage, superior safety profile and arguably a smoother regulatory landscape^17,18^, constituting a promising therapeutic alternative.

In the present study, we investigated IFP-MSC EXOs protein and miRNA signatures, and their effects on stimulated with inflammatory and fibrotic cues, synoviocytes, and macrophages. IFP-MSC show potent molecular and protein immunomodulatory exosomal profiles whereas IFP-MSC EXOs significantly affect synoviocytes and macrophages functionality in inflammatory conditions in vitro. Importantly, in an acute synovial/IFP inflammation rat model, therapeutic treatment with IFP-MSC EXOs promote robust macrophage polarization toward an anti-inflammatory therapeutic M2 phenotype within the synovium/IFP. These types of observations could provide a rationale for further testing of the IFP-MSC EXOs as a viable therapeutic modality to manufacture cell-free products for both synovitis/IFP fibrosis as well as chronic conditions such as osteoarthritis (OA) where inflammation is increasingly recognized as an important component of the disease pathology.

## Results and discussion

### IFP-MSC EXOs isolation and characterization

Our previous studies clearly demonstrated that IFP-MSC manufacturing under regulatory-compliant conditions (human platelet lysate—hPL) results in a fine-tuned, regulatory-compliant, cell-based product with superior immunomodulatory properties that overcome any donor-to-donor variability in biological functionality^19–21^. On this basis, hPL-expanded IFP-MSC can efficiently degrade substance P and suppress T cell proliferation in vitro, whereas they can dramatically reverse signs of synovitis and IFP fibrosis upon intra-articular infusion in vivo^20,21^. Therefore, herein using our established IFP-MSC manufacturing protocol we investigated IFP-MSC EXOs protein and miRNA signatures, and their immunomodulatory/anti-inflammatory functionality in vitro.

**Figure 1:**
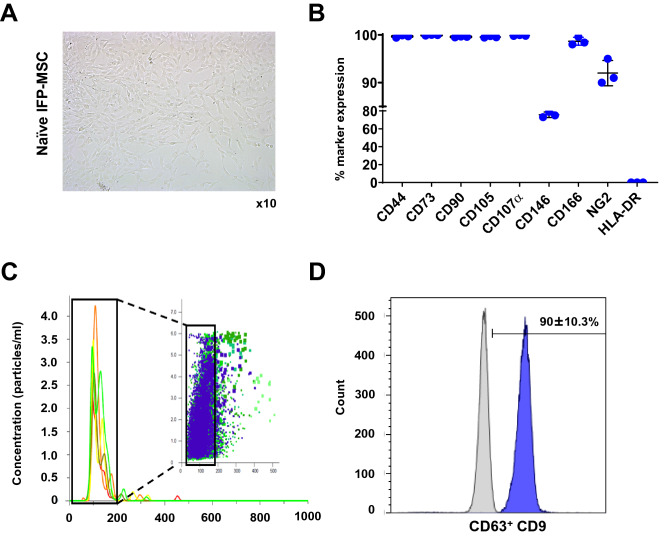
IFP-MSC culture and IFP-MSC EXOs isolation. (**A**, **B**) IFP-MSC showed a fibroblast-like morphology and high expression (> 95%) of common MSC-defining markers. Interestingly, IFP-MSC had high expression of CD146 (77.0 ± 3.0%) and NG2 (92.5 ± 2.7%) pericytic markers, whereas showed absence of human leukocyte antigen HLA-DR (n = 3). (**C**) Nanoparticle tracking analysis showed that isolated vesicles from IFP-MSC conditioned medium are < 200 nm diameter size, consistent with the known size of exosomes. (**D**) CD63^+^ -selected exosomes showed purity by high positivity for exosomal marker CD9.

As expected, IFP-MSC cultured in Ch-R medium showed fibroblast-like morphology (Fig. 1A) and high expression (> 95%) of common MSC-defining markers as established by ISCT^22,23^, including CD44, CD73, CD90, CD105, and CD107α (Fig. 1Β). Similar to previous reports^24^, the CD146 and NG2 markers which define the pericytic origin of MSC^25,26^, were highly expressed (77.0 ± 3.0% and 92.5 ± 2.7%, respectively). Importantly, human leukocyte antigen HLA-DR was absent in all IFP-MSC donors. These data indicate that IFP-MSC retain their perivascular MSC-related immunophenotype without signs of immunogenicity in vitro.

IFP-MSC EXOs were characterized by size and surface marker expression following the guidelines recommended by ISEV (International Society for Extracellular Vesicles), termed MISEV2018 (Minimal Information for Studies of Extracellular Vesicles 2018)^27^ (Fig. 1C). Nanoparticle tracking analysis showed that isolated vesicles from IFP-MSC conditioned medium are < 200 nm diameter size, consistent with the known size of exosomes. IFP-MSC EXOs yield was 3–4 × 10^9^/ml as detected by nanoparticle tracking analysis of all IFP-MSC donors. Also, CD63^+^-selected exosomes showed purity by high positivity for exosomal marker CD9 (90.0 ± 10.3%) (Fig. 1D).

### miRNA and protein signature of IFP-MSC EXOs

MicroRNAs (miRNAs) are single-stranded RNA molecules that can influence cell functionality by binding to the 3′-UTR of cognate mRNAs, leading to the degradation or translational inhibition of target mRNAs^28,29^. Studies have demonstrated their immunomodulatory roles and their potential for inflammatory disease treatment^30^. In parallel, exosomes with specific miRNA cargos can be isolated from various joint tissues including infrapatellar fat pad that can influence whole joint homeostasis^31^. Based on this, one study demonstrated that IFP-MSC EXOs highly abundant in miR-100-5p can effectively ameliorate gait abnormalities in OA mice and alleviate articular cartilage lesions in vivo^32^.

**Figure 2:**
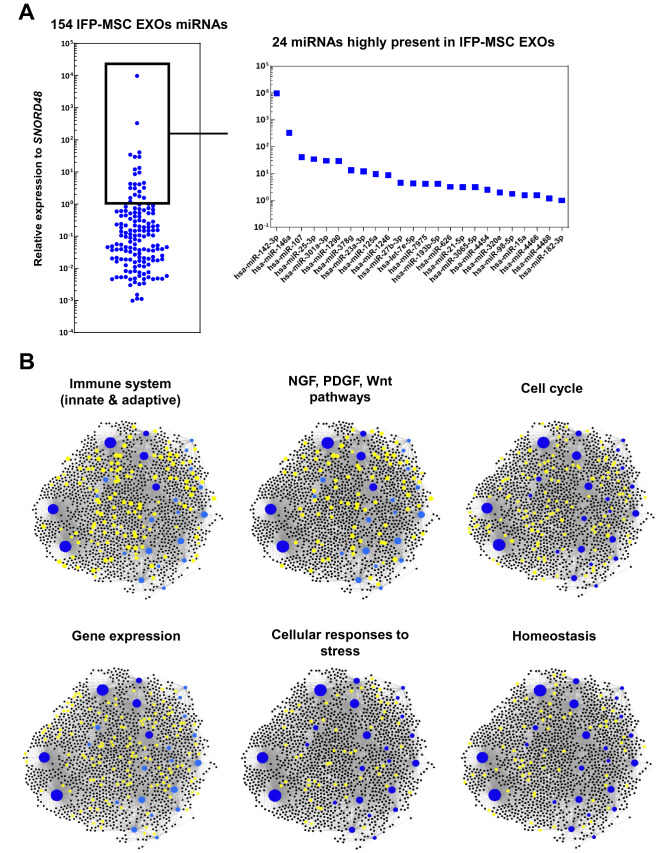
The miRNA signature of IFP-MSC EXOs. (**A**) 154 miRNAs were detected as cargo within IFP-MSC EXOs with 24 miRNAs being highly present. (**B**) Reactome analysis of 24 miRNAs highly present in exosomes showed their involvement in the regulation of six gene groups related to the: immune system, NGF/PDGF/Wnt pathways, cell cycle, gene expression, cellular responses to stress, and homeostasis.

Herein, we investigated the MSC-related miRNA cargo of IFP-MSC EXOs cultured in Ch-R medium. From 166 MSC-related miRNAs assessed, 154 were present as cargo within IFP-MSC EXOs with 24 miRNAs being highly present (Fig. 2A). Furthermore, the first 4 out of 24 highly present miRNAs (hsa-miR-142-3p, hsa-miR-146a, hsa-miR-107, hsa-miR-25-3p) show strong involvement in immune cell regulation. Interestingly, studies have shown that hsa-miR-142-3p attenuates phagocytosis and cytokine secretion of inflammatory mediators, including TNF-α, IL-6, and IL-12p40, in myeloid inflammatory cells whereas it relieves neuropathic pain by targeting high mobility group box 1^33,34^. The second highly present miRNA cargo, hsa-miR-146a, belongs to the hsa-miR-146 family of genes that are expressed in response to pro-inflammatory stimuli as negative feedback to control excessive inflammation. Specifically, studies showed that hsa-miR-146a negatively regulates the adaptive immunity by modulating adaptor protein (AP)-1 activity and IL-2 expression in T cells, as well as immune cell activation and cytokine production^35,36^. In addition, the expression levels of hsa-miR-107 have been demonstrated to be related to TLR4 activation whereas hsa-miR-107 decreased expression may be a regulative feedback effect to limit insulin resistance in inflammation^37^. Studies have shown that has-miR-25-3p inhibits macrophage secretion of inflammatory cytokines in sepsis by targeting high mobility group box 1 whereas MSC EXOs cargo alleviates myocardial infarction by targeting pro-apoptotic proteins in vivo^38,39^. In the present study, reactome analysis of 24 miRNAs highly present in exosomes showed their involvement in the regulation of six gene groups related to the: immune system, NGF/PDGF/Wnt pathways, cell cycle, gene expression, cellular responses to stress, and homeostasis (Fig. 2B and Table 1). Overall, from a clinical standpoint the potent functionality of these highly present miRNAs can strongly support the notion of developing novel cell-free therapeutics for inflammation/fibrosis reversal based on MSC secretome. Furthermore, miRNA interactome analysis revealed 6 miRNAs (hsa-miR-98-5p, hsa-let-7e-5p, hsa-miR-21-5p, hsa-miR-15a-5p, hsa-miR-25-3p, hsa-miR-107) with higher node degree that act as hubs in a gene network (Supplementary Fig. 1). Even though the levels of these miRNAs as cargo within the IFP-MSC EXOs are variable, they control multiple genes related to important pathways such as Jak-STAT signaling pathway, focal adhesion, neurotrophin signaling pathway, p53 signaling pathway, cytokine-cytokine receptor interaction and others.

**Table 1:** Reactome analysis of 24 miRNAs highly present in IFP-MSC EXOs.

Top reactome hits	Number of genes	Pval
Gene expression	134	1.12E−09
Immune system	123	0.00015
Innate immune system	79	0.000428
Cell cycle	67	0.00268
Hemostasis	63	0.00135
Adaptive immune system	62	0.000691
Cellular responses to stress	55	6.18E−09
Signaling by NGF	52	9.64E−07
NGF signaling via TRKA from the plasma membrane	41	4.33E−07
Generic transcription pathway	41	4.33E−07
Signaling by PDGF	40	1.90E−07
Signaling by Wnt	40	0.000578

**Figure 3:**
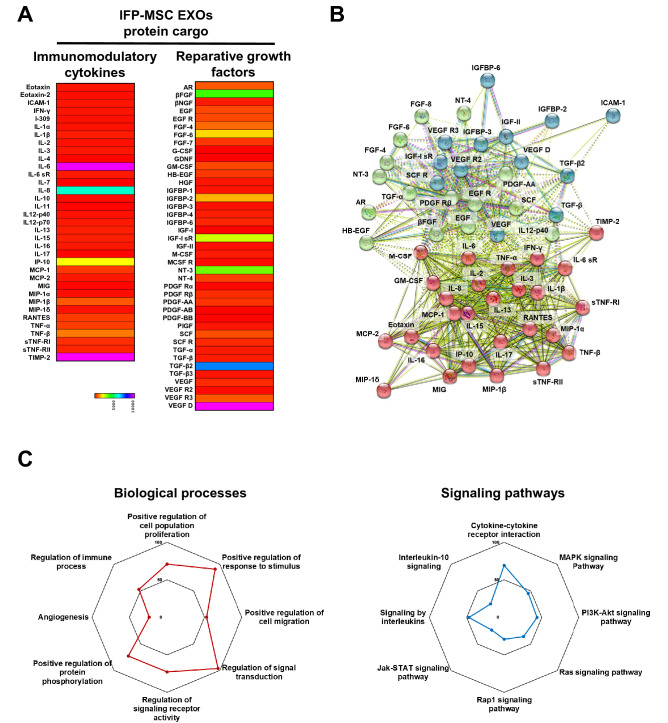
Protein signature of IFP-MSC EXOs. (**A**) IFP-MSC EXOs were enriched for immunomodulatory and reparative proteins. (**B**) All proteins appeared interconnected at least through one association and into three groups (red, green, blue). All K-means clustering networks demonstrated elevated protein–protein interaction (PPI) enrichment (*p* < *1.0e−16*) and an average local clustering coefficient > 0.7 indicating that the proteins used are at least partially biologically connected. (**C**) Identified proteins are involved in multiple biological processes and pathway regulation in KEGG reactome analysis.

We detected multiple immunomodulatory and reparative molecules secreted as a cargo within the IFP-MSC EXOs (Fig. 3A). The exosomal “signature” involves the upregulated secretion of key immunomodulatory molecules including IL-6, IL-8, IP-10, TIMP-2. IL-6 and IL-8 cytokines have been implicated in both pro- and anti-inflammatory actions with previous studies indicating that IL-6/IL-8 ratio plays a crucial role in the specific polarization of the cellular microenvironment^40^. Importantly, studies have illustrated that IP-10 secretion directly correlates with decreased T cell proliferation^41^ whereas TIMP-2 attenuates the development of inflammation and inflammatory pain via MMP-dependent and receptor-mediated cell signaling mechanisms^42^. Specifically, NF-κB, Nrf2, and CREB pathways are largely involved in TIMP-2-mediated anti-inflammatory effects^43^. In parallel, key reparative molecules including TGF-β2, VEGFD, βFGF, IGF-I sR showed upregulated secretion in IFP-MSC EXOs. TGF-β2 is a key molecule affecting cell proliferation, differentiation and apoptosis via various signal transduction networks such as serine/threonine receptors and SMAD protein phosphorylation. Of note, studies showed that TGF-β2 can suppress IL-2-dependent T cell proliferation^44^. Interestingly, VEGF secreted by MSC induces endothelial progenitor cell differentiation towards endothelial cells via paracrine actions^45^.

All proteins appeared interconnected at least through one association and into three groups (red, green, blue, Fig. 3). All K-means clustering networks demonstrated elevated protein–protein interaction enrichment (*p* < *1.0e−16*) and an average local clustering coefficient > 0.7 indicating that the proteins used are at least partially biologically connected (Fig. 3B). In terms of biological processes, various categories were highly affected including regulation of signal transduction, positive regulation of response to stimulus, regulation of signaling receptor activity, positive regulation of protein phosphorylation, positive regulation of cell population proliferation, positive regulation of cell migration, regulation of immune process, and angiogenesis. Combined, these effects empower IFP-MSC EXOs to respond to inflammation/injury by altering key cascades known to affect local immune responses. Regarding the type of signaling pathways affected, IFP-MSC EXOs have profound effects on cytokine-cytokine receptor interaction, signaling by interleukins, MAPK signaling, PI3K-Akt signaling pathway, Ras signaling, Rap1 signaling, Jak-STAT signaling, and interleukin-10 signaling (Fig. 3C). Both cytokine-cytokine receptor interaction and signaling by interleukins which are among the highest affected pathways play crucial role in regulating inflammatory and pain cascades. Similarly to IFP-MSC EXOs protein cargo, our previous published data showed that IFP-MSC soluble protein secretomes have significant involvement in cytokine-cytokine receptor interaction and signaling by interleukins^20^.

Therefore, both miRNA and protein cargo of IFP-MSC EXOs strongly indicate their potent functionality which upon further studies could result in the development of specialized cell-free therapies that overcome regulatory constrains for safe and effective regulation of inflammation and fibrosis in humans.

### IFP-MSC EXOs effects on synoviocytes and macrophages

The histological profile of the synovium in OA preclinical models^13^ and in OA patients^46^ is mainly characterized by synovial lining hyperplasia and IFP fibrosis. The main cellular types contributing to these histological changes are synoviocytes and macrophages. Specifically, synoviocytes can be divided into type A (macrophage-like synoviocytes) which constitute resident macrophages, and type B (fibroblast-like synoviocytes) which display typical fibroblast markers such as surface marker Thy-1 (CD90) and are mainly responsible for maintenance of synovial homeostasis^47,48^. We separately investigated the effects of two IFP-MSC EXOs concentrations (EXO and 10 × EXO) on naïve and stimulated synoviocytes in vitro in an effort to identify the optimal therapeutic dosage for in vivo application.

**Figure 4:**
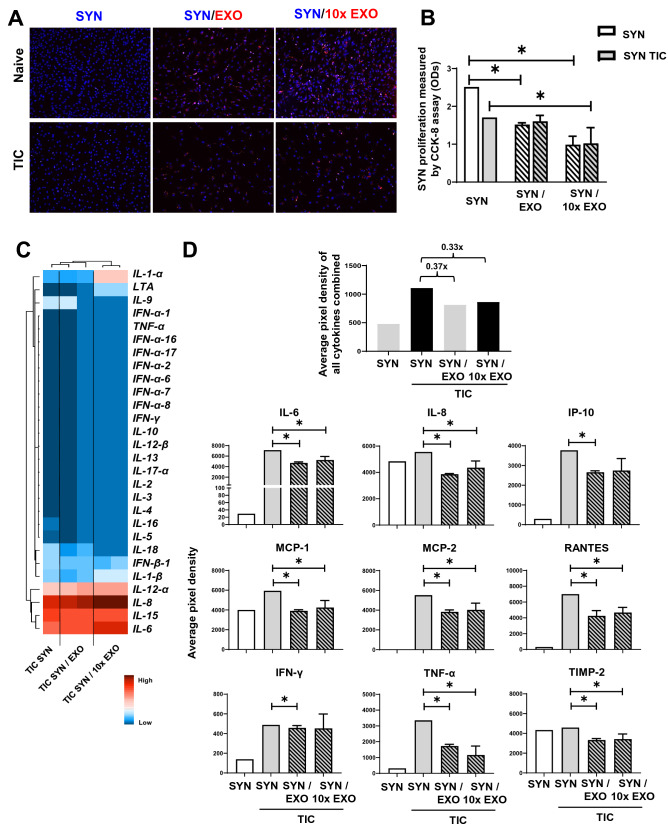
IFP-MSC EXOs anti-inflammatory effects on synoviocytes. (**A**) IFP-MSC EXOs were internalized in both naïve and TIC-primed SYN (blue, nucleus; red, EXOs). (**B**) SYN and TIC SYN proliferation was attenuated by both EXO and 10 × EXO. (**C**) TIC SYN inflammation-related transcriptional profiling was altered by IFP-MSC EXOs. (**D**) IFP-MSC EXOs significantly reduced secretion of pro-inflammatory molecules from TIC SYN compared to SYN stimulated alone (*p < *0.05*).

In co-cultures, IFP-MSC EXOs were internalized in both naïve and TIC-primed SYN whereas SYN and TIC SYN proliferation was attenuated by both EXO and 10 × EXO (Fig. 4A,B). This is an important finding suggesting to us that IFP-MSC EXOs could attenuate synovial lining hyperplasia upon their in vivo intra-articular infusion. Similarly, a previous study showed that synoviocytes exposure to BM-MSC EXOs results in suppressed rheumatoid arthritis-synoviocyte activation, migration, and invasion in vitro^49^. At the molecular level the TIC-SYN inflammation-related profile was altered by IFP-MSC EXOs. Of note, increased expression of *IL-1-α* and *IL-6* may be counter balanced by increased *IL-8* expression levels (Fig. 4C). IL-8 has been recognized to have anti-inflammatory activity, which has been established in various inflammation models. These molecular data are coupled by SYN secretory protein profiling in SYN/ IFP-MSC EXOs co-cultures. Overall, the secretion of immunomodulatory molecules was reduced 3.7-fold for EXO and 3.3-fold for 10 × EXO. Specifically, IL-6, IL-8, IP-10, MCP-1, MCP-2, RANTES, IFN-γ, TNF-α, TIMP-2 were significantly (*p* < *0.05*) reduced upon exposure to IFP-MSC EXOs compared to SYN stimulated alone (Fig. 4D). These findings indicate the strong capacity of the IFP-MSC secretome to attenuate not only the proliferation but also the pro-inflammatory activation of type A and type B synoviocytes in vitro.

In patients with OA, both the IFP and the synovium are infiltrated by various immune cells including monocytes/macrophages. These infiltrates complement the resident cells with macrophage polarization towards the pro-inflammatory classical M1 phenotype (reviewed in^48^). Specifically, in patients with OA, macrophages are recruited within the synovium in increased numbers compared to non OA patients and form clusters of multinucleated giant cells. Of note, studies have demonstrated a significant association between multinucleated giant cell numbers and synovitis severity^46^. On this basis, macrophage phenotype manipulation has been proposed as a potential therapy given that initial data indicate polarization of macrophages towards an alternative anti-inflammatory M2 phenotype can be induced. Herein, we investigated the effects of two IFP-MSC EXOs concentrations (EXO and 10 × EXO) on naïve and stimulated macrophages in vitro.

**Figure 5:**
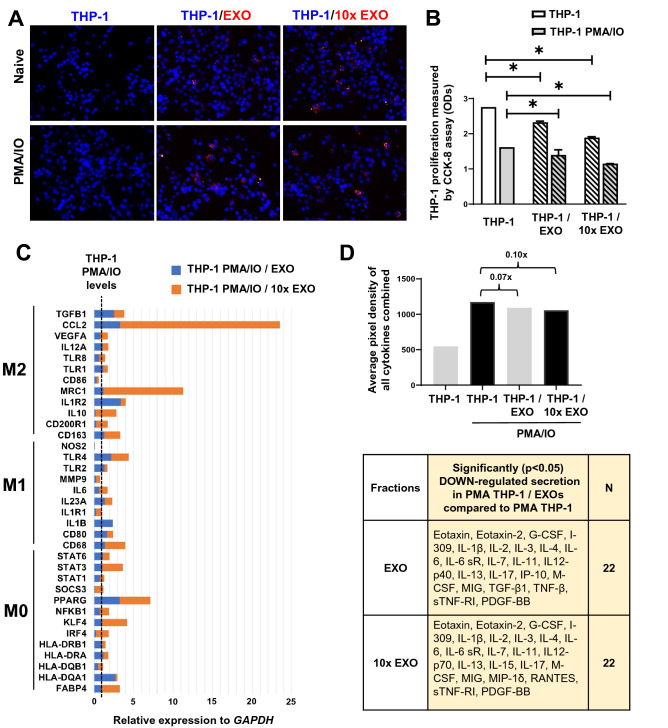
IFP-MSC EXOs immunomodulatory effects on macrophages. (**A**) IFP-MSC EXOs were internalized in both naïve and PMA/IO-stimulated THP-1 macrophages (blue, nucleus; red, EXOs). (**B**) THP-1 and PMA/IO-stimulated THP-1 proliferation was attenuated by both EXO and 10 × EXO. (**C**) PMA/IO-stimulated THP-1 molecular profiling indicated a strong gene expression shift towards an M2 macrophage polarization by both EXO and 10 × EXO. (**D**) IFP-MSC EXOs significantly reduced secretion of pro-inflammatory molecules from PMA/IO-stimulated THP-1 compared to stimulated macrophages alone (*p < *0.05*).

Similar to synoviocytes, naïve and PMA/IO-stimulated THP-1 macrophages didn’t only internalized IFP-MSC EXOs, but also attenuated their proliferation in both EXO and 10 × EXO concentrations (Fig. 5A,B). At the molecular level, PMA/IO-stimulated THP-1 molecular profiling indicated a strong gene expression shift towards an M2 macrophage polarization by both EXO and 10 × EXO (Fig. 5C). Most importantly, the expression levels of *MRC1* (*Cd206*) characteristic M2-polarization macrophages marker^50^ was strongly induced when macrophages were exposed to 10 × EXO. *CCL2* expression was also significantly induced in 10 × EXO. CCL2 chemokine is associated with monocyte recruitment in inflamed tissues via CCR2 chemokine receptor after pro-inflammatory cytokines activation. Importantly, CCL2 and CCR2 determine the extent of M2 macrophage polarization by enhancing the production of anti-inflammatory IL-10 cytokine^51^. Overall, the secretion of immunomodulatory molecules was reduced 0.07x for EXO and 0.1x for 10 × EXO. Specifically, we observed a significant (*p* < *0.05*) down-regulated secretion of 22 immunomodulatory proteins for both EXO and 10 × EXO in PMA THP-1/EXO compared to PMA THP-1 (Fig. 5D). These findings indicate the significant effect of IFP-MSC EXOs on macrophages at both the molecular and protein levels. Collectively, these data are consistent with our previous reports indicating an M1 to M2 IFP macrophage phenotypic polarization following a single intra-articular injection of a subset of BM-MSC in rats with induced synovitis and IFP fibrosis^52^.

### IFP-MSC EXOs effects on synovitis, IFP fibrosis and macrophages polarization in vivo

**Figure 6:**
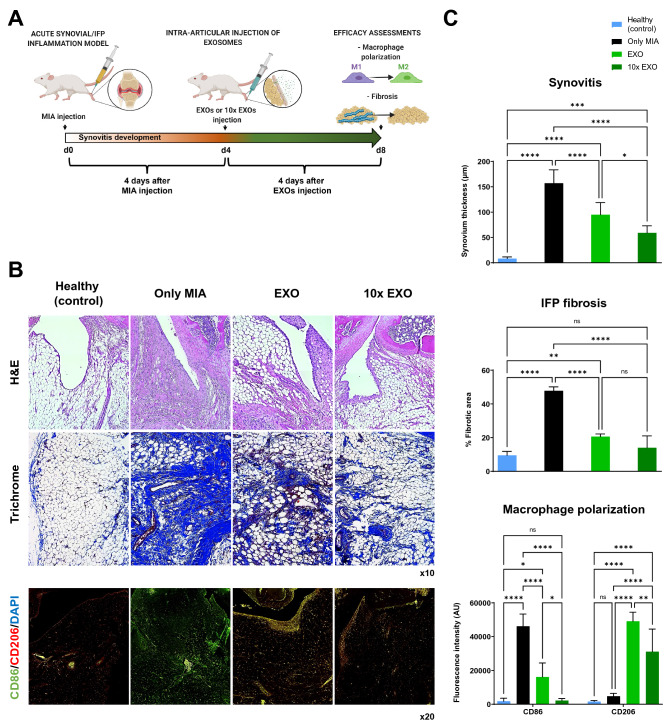
IFP-MSC EXOs effectively reverse synovitis/IFP fibrosis and polarize macrophages towards an alternatively activated-M2 phenotype in vivo. (**A**) Schematic indicating the generation of acute synovitis/IFP fibrosis rat model, IFP-MSC EXOs therapeutic intervention and chronological evaluation. (**B**,**C**) Hematoxylin/Eosin staining (top panel), Masson’s trichrome staining (middle panel) and CD86/CD206 immunolocalization (lower panel) in sagitally-sectioned knees of representative rats for healthy (control), diseased (only MIA) or IFP-MSC treated (with EXO and 10 × EXO). Compared with only MIA injected group, which showed significant synovitis/IFP fibrosis and largely M1 (CD86^+^) macrophages polarization, a striking correlation was found between IFP-MSC EXOs treatment and synovitis/fibrosis attenuation, and macrophages polarization towards alternatively activated-M2 (CD206^+^) phenotype (ns: non-significant, *p < 0.05, **p < 0.01, ***p < 0.001, ****p < *0.0001*).

An acute synovial/IFP inflammation rat (MIA) model was used to test the capacity of IFP-MSC EXOs upon intra-articular infusion to reverse synovial and IFP inflammation, IFP fibrosis, and macrophage polarization from M1 to M2 phenotypes (Fig. 6A).

Compared to healthy rat knees, the diseased group (only MIA) demonstrated strong synovitis (157.0 ± 26.5 μm synovium thickness), characterized by synovial lining hyperplasia and IFP fibrosis (47.8 ± 2.3% of fibrotic area) and characterized by loss of adipocytes within the main body of the IFP tissue (Fig. 6B,C). According to Udo et al. infrapatellar fat pad inflammation scoring (0–5) for rat arthritis in the MIA model^53^, only the diseased group developed grade 3 synovitis and grade 2 to 3 IFP fibrosis. Upon EXO or 10 × EXO infusion, synovitis was significantly (*p* < *0.05*) reduced (95.0 ± 23.9 μm and 59.1 ± 13.8 μm synovium thickness, respectively) compared to the diseased group. Similarly, both EXO and 10 × EXO infusion resulted in increased (*p* < *0.05*) fibrosis clearance from the IFP main body (20.6 ± 1.6% and 14.0 ± 7.0% of fibrotic area, respectively). Interestingly, a previous study indicated that fibroblast-like synoviocytes contribute to fibrogenesis by excessive extracellular matrix deposition and enhanced contractile function^54^. Therefore, upon IFP-MSC EXOs infusion reduction of synovitis may be directly related to reduced IFP fibrosis in vivo.

Synovium/IFP resident and infiltrated macrophages have demonstrated key roles in joint homeostasis and articular cartilage degeneration, as they are the source of inflammatory and catabolic mediators such as IFN-γ, TNF-α, IL-1, IL-6, IL-8, and metalloproteinases^48,55^. On this basis, we performed classically activated-M1 (CD86^+^)/alternatively activated-M2 (CD206^+^) macrophage phenotypic evaluations to determine mechanistic cellular changes promoted in synovium/IFP tissues after intra-articular IFP-MSC EXO infusion. Importantly, previous studies showed that parent MSCs can effectively modulate macrophage polarization in vivo^52,56^. In the present study, MIA infusion resulted in a significant (*p* < *0.05*) shift of macrophage polarization towards the M1 phenotype (M1/M2 ratio: 9.6) from the homeostatic status of IFP tissue in healthy group (M1/M2 ratio: 1.1). Four days after IFP-MSC EXOs infusion, macrophage polarization was adversely shifted towards the alternatively activated-M2 phenotype (M1/M2 ratio: 0.3), whereas the M1 phenotype was significantly (*p* < *0.05*) reduced compared to the MIA group only (16,134 vs 46,179 AUs, respectively). Remarkably, 10 × EXO infusion resulted in almost ten times stronger macrophage polarization towards the M2 phenotype (M1/M2 ratio: 0.07) compared to EXOs, with M1-polarized macrophage subpopulation reverting to homeostatic conditions (2,212 AUs in 10 × EXO and 1,790 AU in healthy groups). These data are in accordance with a previous study indicating that a single intra-articular infusion of adipose tissue-derived MSC EXOs not only inhibits M1 macrophage infiltration into the synovium but also protects from articular cartilage degeneration^57^. Importantly, our present cell-free approach further reinforces our previous single-cell RNA-sequencing data which clearly demonstrated that macrophages within the IFP can shift towards a more immunomodulatory polarization status after a single IFP-MSC intra-articular therapy^14^. Therefore, both EXO and 10 × EXO treatments suggest robust macrophage polarization toward an anti-inflammatory therapeutic M2 phenotype within the synovium/IFP tissues.

## Conclusions

In summary, IFP-MSC show potent molecular and protein immunomodulatory exosomal profiles that significantly affect synoviocytes and macrophages functionality in inflammatory conditions in vitro. Our results help elucidate various local activities of IFP-MSC EXOs within the IFP during joint disease pathogenesis and progression. However, this study has some limitations that deserve consideration. Specifically, the effects of IFP-MSC EXOs on T and B cell proliferation and maturation should be investigated in further experiments. Importantly, from a therapeutic perspective the local activities of intra-articularly infused EXOs may be related not only with the control of IFP fibrosis, but also with robust immune-related responses via shift of M1 to M2 macrophages polarization status. Therefore, based on our findings, we propose a proof-of-concept viable cell-free alternative to MSC-based therapeutics in the treatment of joint disease characterized by synovitis, IFP fibrosis, articular cartilage degradation, and potentially OA. Further studies are needed to strongly support the robust IFP-MSC EXOs therapeutic effects in various joint OA phenotypes.

## Materials and methods

### Isolation, culture and expansion of IFP-MSC

All experiments using human cells were performed in accordance with relevant guidelines and regulations. Human IFP-MSC were isolated from IFP tissue obtained from de-identified, non-arthritic patients that were age- and sex-matched (n = 5; three males and two females; 36.6 ± 12.9 years old) undergoing elective knee arthroscopy at the Lennar Foundation Medical Center at the University of Miami. Informed consent was obtained from all participants and/or their legal guardians. All procedures were carried out following approval by the University of Miami IRB not as human research (based on the nature of the samples as discarded tissue). IFP tissue (5–10 cc) was mechanically dissected and washed repeatedly with Dulbecco’s Phosphate Buffered Saline (DPBS; Sigma Aldrich, St Louis, MO, USA), followed by enzymatic digestion using 235 U/ml Collagenase I (Worthington Industries, Columbus, OH, USA) diluted in DPBS and 1% bovine serum albumin (Sigma) for 2 h at 37 °C with agitation. Enzymatic digestion was inactivated with complete media with DMEM low glucose GlutaMAX (ThermoFisher Scientific, Waltham, MA, USA) containing 10% fetal bovine serum (FBS; VWR, Radnor, PA, USA), washed and seeded at a density of 1 × 10^6^ cells/175 cm^2^ flask in chemically-reinforced (Ch-R) Mesenchymal Stem Cell Growth Medium 2 (PromoCell, Heidelberg, Germany). Forty-eight hours (48 h) post-seeding, non-adherent cells were removed by DPBS gentle rinsing and fresh media were replenished accordingly.

All MSC were cultured at 37 °C 5% (v/v) CO_2_ until 80% confluent as passage 0 (P0), then passaged at a 1:5 ratio until P3, detaching them with TrypLE™ Select Enzyme 1X (Gibco, ThermoFisher Scientific) and assessing cell viability with 0.4% (w/v) Trypan Blue (Invitrogen, ThermoFisher Scientific).

### Immunophenotype

Flow cytometric analysis was performed on P3 IFP-MSC (n = 3). Briefly, 2.0 × 10^5^ cells were labelled with monoclonal antibodies specific for: CD44, CD73, CD90, CD105, CD107α (Biolegend, San Diego, CA), CD146 (Miltenyi Biotec, Auburn, CA), CD166, NG2, HLA-DR (BD Biosciences, San Jose, CA), and the corresponding isotype controls. All samples included a Ghost Red Viability Dye (Tonbo Biosciences, San Diego, CA, USA). Data (20.000 events collected) were acquired using a Cytoflex S (Beckman Coulter, Brea, CA, USA) and analyzed using Kaluza analysis software (Beckman Coulter).

### Isolation and validation of IFP-MSC derived exosomes

IFP-MSC derived exosomes (IFP-MSC EXOs) were isolated from IFP-MSC conditioned medium by a step-wise ultracentrifugation method and CD63-immunomagnetic purification. Briefly, conditioned media from IFP-MSC groups cultured in exosome-depleted Ch-R medium^58^ are filtered through a 0.22 µm filter to remove debris and large vesicles, and differentially centrifuged for 2000×*g* for 10 min, 10,000×*g* for 30 min, and ultracentrifuged for 120,000×*g* for 16hr^59^. Pre-enriched exosome preparations were incubated with the Dynabeads®-based Exosome-Human CD63 Isolation/Detection Reagent (Invitrogen) and using a magnetic separator, exosome preparations are further purified. Samples from each group were assessed for biophysical and biochemical characterization^60^.

Quantity and size determination were performed by nanoparticle tracking analysis (NTA) (NanoSight NS300, Malvern). Samples were diluted in PBS from 1:10 to 1:100 depending on the initial sample concentration. The software settings for analysis were: detection threshold 5; room temperature; number of frames 30 and measurement time 30 s. The size distribution and particle concentration each represent the mean of five individual measurements.

Exosomes presence was validated by CD9 (Invitrogen) expression in CD63^+^-gated particles by flow cytometry. Data (20.000 events collected) were acquired using a Cytoflex S (Beckman Coulter) and analyzed using Kaluza analysis software (Beckman Coulter).

The functional assessment of IFP-MSC EXOs was performed in two concentrations corresponding to exosomes secreted from 5 × 10^5^ (EXO) and 5 × 10^6^ (10 × EXO) IFP-MSC. For IFP-MSC EXOs tracking, exosomes were stained with PKH26 red fluorescent membrane staining kit (Fluorescent Cell Linker Kits, Sigma) according to manufacturer’s instructions and co-cultured with target cells in functional assessments.

### miRNA profile of IFP-MSC EXOs

miRNA and protein were extracted from IFP-EXOs using Total Exosome RNA and Protein Isolation Kit (Thermo Fisher Scientific) according to manufacturer’s instructions. Total exosome miRNA (1 μg) was used for first-strand cDNA synthesis with All-in-One miRNA First-Strand cDNA Synthesis Kit (GeneCopoeia, Rockville, MD).

Pre-designed human MSC exosome 166 miRNA qPCR arrays (GeneCopoeia) were performed using 1000 ng cDNA per IFP-MSC sample (n = 2), and processed using StepOne Real-time thermocycler (Applied Biosystems, LLC). Data analysis was performed using qPCR result with GeneCopoeia’s online Data Analysis System (http://www.genecopoeia.com/product/qpcr/analyse/). Mean values were normalized to small nucleolar RNA, C/D box 48 (SNORD48), expression levels were calculated using the 2^−ΔCt^ method. Putative miRNA interactomes were generated using a miRNet centric network visual analytics platform (https://www.mirnet.ca/). The miRNA target gene data were collected from well-annotated database miRTarBase v8.0 and miRNA-gene interactome network refining was performed with 2.0 betweenness cut-off. Values (with 34 cycles cut-off point) were represented in a topology miRNA-gene interactome network using force atlas layout and hypergeometric test algorithm. InfoMap algorithm was also used to identify miRNAs with higher node degree that act as hubs in a gene network.

### Protein profile of IFP-MSC EXOs

Multiplex protein arrays of 35 cytokines and 41 growth factors (RayBio® C-Series, RayBiotech, Peachtree Corners, GA) were used to determine IFP-MSC EXOs protein cargo (n = 2). One mL of IFP-MSC EXOs protein extract was used for each assay following the manufacturer’s instructions. Data shown represent 40 s exposure in FluorChem E chemiluminescence imaging system (ProteinSimple, San Jose, CA). Results were generated by quantifying the mean spot pixel density of each array using protein array analyser plugin using ImageJ software (Fiji/ImageJ, NIH website). The signal intensities were normalized with the background whereas separate signal intensity results represent the average pixel density of two spots per protein. Signal intensity for each protein spot is proportional to the relative concentration of the antigen in the sample.

### Pathway analysis

Putative interactomes were generated by Search Tool for Retrieval of Interacting Genes/Proteins (STRING 11.0; available from: http://string-db.org) database using interaction data from experiments, databases, neighbourhood in genome, gene fusions, co-occurrence across genomes, co-expression and text-mining. An interaction confidence score of 0.4 was imposed to ensure high interaction probability. K-means clustering algorithm was used to organize proteins into 3 separate clusters per condition tested, discriminated by colours. Venn diagrams were used to demonstrate all possible relations between naïve and/or TIC-primed IFP-MSC cultured under all three different conditions for the significantly (*p* < *0.05*) altered proteins. Functional enrichments related to biological process, Kyoto Encyclopedia of Genes and Genomes (KEGG) pathways, and reactome pathways were presented in radar graphs for proteins tested.

### Synoviocyte inflammation assay

Passage 1 synoviocytes (SYN) expanded in synoviocyte medium (ScienCell, Carlsbad, CA) were subsequently primed with TIC inflammatory/fibrotic cocktail (15 ng/ml TNFα, 10 ng/ml IFNγ, 10 ng/ml CTGF) for 72 h. On day 3, synoviocyte/exosome (SYN/ IFP-MSC EXOs) co-cultures were performed using EXO and 10 × EXO for each sample. Co-cultures were fed with synoviocyte medium or synoviocyte medium + TIC inflammatory/fibrotic cocktail for 72 h according to the initial non-induced or TIC-primed cohorts designation.

CCK-8 cytotoxicity assay (Cell Counting Kit-8, Sigma) was performed in non-induced or TIC-primed SYN and SYN/ IFP-MSC EXOs according to manufacturer’s instructions. EXO and 10 × EXO cytotoxicity was determined by measuring optical densities of individual wells at 450 nm (SpectraMax M5 spectrophotometer, Molecular Devices, San Jose, CA).

RNA extraction from SYN cultures was performed using the RNeasy Mini Kit (Qiagen, Frederick, MD) according to manufacturer’s instructions. Total RNA (1 μg) was used for reverse transcription with SuperScript™ VILO™ cDNA synthesis kit (Invitrogen). A pre-designed 28 gene Taqman low density cytokine array (TLDA, Applied Biosystems) was performed using 1000 ng cDNA per culture and processed using StepOne Real-time thermocycler (Applied Biosystems, LLC). Data analysis was performed using DataAssist software v2.0 (Applied Biosystems, LLC). Mean values were normalized to GAPDH, expression levels were calculated using the 2^−ΔCt^ method. Values were represented in a fold change heatmap as the relative fold change of the TIC SYN/EXO or TIC SYN/10 × EXO to TIC SYN (reference sample, 2^−ΔCt^ = X sample/X reference sample).

Secretory profiles of non-induced and TIC-primed cohorts were evaluated using multiplex protein arrays of 40 cytokines (RayBio® C-Series, RayBiotech). One mL of conditioned media was used for each assay following the manufacturer’s instructions. Data shown represent 40 s exposure in FluorChem E chemiluminescence imaging system (ProteinSimple, San Jose, CA). Results were generated by quantifying the mean spot pixel density of each array using protein array analyser plugin using ImageJ software (Fiji/ImageJ, NIH website). Signal intensities were normalized with the background whereas separate signal intensity results represent the average pixel density of two spots per protein. The signal intensity for each protein spot is proportional to the relative concentration of the antigen in the sample.

### Macrophage polarization assay

Human monocytes (THP-1, ATCC) were differentiated into macrophages using PMA/IO (Phorbol 12-myristate 13-acetate/Ionomycin) and polarized to M1 macrophages by M1-macrophage generation medium (PromoCell). PMA/IO-stimulated THP-1/IFP-MSC EXOs co-cultures were performed for 2 days using EXO and 10 × EXO. Generated macrophages inflammation-related protein profiling was performed by multiplex protein arrays whereas their polarization status was assessed using macrophage polarization qPCR array (ScienCell).

A CCK-8 cytotoxicity assay (Cell Counting Kit-8, Sigma) was performed in non-induced or PMA/IO-primed THP-1 and THP-1/IFP-MSC EXOs according to manufacturer’s instructions. EXO and 10 × EXO cytotoxicity was determined by measuring optical densities of individual wells at 450 nm (SpectraMax M5 spectrophotometer, Molecular Devices, San Jose, CA).

Secretory profiles of PMA/IO-primed cohorts were evaluated using multiplex protein arrays of 40 cytokines (RayBio® C-Series, RayBiotech). One mL of conditioned media was used for each assay following the manufacturer’s instructions. Data shown represent 40 s exposure in FluorChem E chemiluminescence imaging system (ProteinSimple, San Jose, CA). Results were generated by quantifying the mean spot pixel density of each array using protein array analyser plugin using ImageJ software (Fiji/ImageJ, NIH website). The signal intensities were normalized with the background whereas separate signal intensity results represent the average pixel density of two spots per protein. The signal intensity for each protein spot is proportional to the relative concentration of the antigen in the sample.

RNA extraction from THP-1 cultures was performed using the RNeasy Mini Kit (Qiagen, Frederick, MD) according to manufacturer’s instructions. Total RNA (1 μg) was used for reverse transcription with SuperScript™ VILO™ cDNA synthesis kit (Invitrogen). A pre-designed 40 gene Human Macrophage Polarization array (GeneQuery™ Human Macrophage Polarization Markers qPCR Array Kit, ScienCell) was performed using 1000 ng cDNA per culture and processed using StepOne Real-time thermocycler (Applied Biosystems, LLC). Mean values were normalized to GAPDH, expression levels were calculated using the 2^−ΔCt^ method. Values were represented in a stacked bar plot for M0, M1, and M2 polarization as the relative fold change of the PMA/IO + THP-1/EXO or PMA/IO + THP-1/10xEXO to PMA/IO + THP-1 (reference sample, 2 − ΔCt = X sample/X reference sample).

### Mono-iodoacetate model of acute synovial/IFP inflammation

All animal experiments were performed in accordance with relevant guidelines and regulations. The animal protocol was approved by the Institutional Animal Care and Use Committee (IACUC) of the University of Miami, USA (approval no. 21–030 LF) and conducted in accordance to the ARRIVE guidelines^61^. Twelve (#12) 10-week old male Sprague Dawley rats (mean weight 250 g) were used. The animals were housed to acclimate for 1 week before the experiment initiation. One rat was housed per cage in a sanitary, ventilated room with controlled temperature, humidity, and under a 12/12 h light/dark cycle with food and water provided ad libitum.

Acute synovial/IFP inflammation was generated by intra-articular injection of 1 mg of mono-iodoacetate (MIA) in 50 µl of saline into the right knee. Briefly, under isoflurane inhalation anesthesia, rat knees were flexed 90º and MIA was injected into the medial side of the joint with a 27G needle using the patellar ligament and articular line as anatomical references. Four (4) days later, a single intra-articular injection of EXO or 10 × EXO in 50 µl of Euro-Collins solution (MediaTech) was performed (similar injection technique), having as control: (1) rat knees receiving MIA but not IFP-EXOs (Only MIA group); and (2) healthy rat knees. Animals were sacrificed at day 4 after IFP-EXOs injection (d8 in total). This short exposure to MIA has been shown to induce inflammatory changes within the synovium and adjacent IFP^53^.

### Cytochemical staining and CD86/CD206 immunolocalization in situ

Rat knee joints were harvested by cutting the femur and tibia/fibula 1 cm above and below the joint line, muscles were dissected and removed, and joints were fixed with 10% neutral buffered formalin (Sigma-Aldrich) for 14 days at room temperature. Knee joints were decalcified, cut along the sagittal plane in half, embedded in paraffin, and serial 4 μm sections obtained. Hematoxylin and Eosin (H&E) staining was performed to evaluate the gross structure and morphology of the knee joints. Masson’s trichrome staining for collagen-rich fibrotic areas was used to evaluate for extent of fibrosis within the fat pad tissue. Microscope images of cytochemically stained tissues were acquired using × 10 objective Leica DMi8 microscope with Leica X software (Leica). Based on histochemical stainings, tissue synovitis/fibrosis was evaluated in 3 rat knees per condition and 4 microscopy fields per knee with Fiji/ImageJ software.

For anti-CD86 and CD206 immunofluorescence staining, sections were incubated with 1 × citrate buffer solution at 60 °C overnight for antigen retrieval, permeabilised with 1 × PBS + 0.2% Triton X-100 for 20 min at room temperature, and incubated with blocking buffer (1 × PBS + 0.1% Triton X-100 with 10% rabbit serum) for 1 h at room temperature. In between different treatments sections were washed with 1 × PBS. Mouse anti-rat CD86 monoclonal antibody and rabbit anti-rat CD206 polyclonal antibody (both from Abcam) were prepared in blocking buffer (1:500) and sections were incubated at 4 °C overnight. Sections were washed with 1 × PBS + 0.01% Triton X-100 and incubated for 2 h with secondary antibodies containing Alexa Fluor488 conjugated goat anti-mouse and Alexa Fluor647 conjugated goat anti-rabbit IgG antibodies (Thermo Fisher Scientific) at room temperature. Controls were incubated with secondary antibodies only. All sections were rinsed with 1 × PBS, mounted in prolong gold antifade reagent with DAPI (Invitrogen), and microscope images were acquired using Leica DMi8 microscope with Leica X software (Leica).

### Statistical analysis

Normal distribution of values was assessed by the Kolmogorov–Smirnov normality test. Statistical analysis was performed using paired and unpaired Student’s *t*-test for normally distributed data and Wilcoxon (for paired data) or Mann Whitney (for unpaired data) test. In the presence of a non-normal distribution of the data, a one-way ANOVA was used for multiple comparisons. All tests were performed with GraphPad Prism v7.03 (GraphPad Software, San Diego, CA). Level of significance was set at *p* < 0.05.

## Supplementary Information

Supplementary Figure S1.

Supplementary Legends.
