# Supplementary figures and images for: Human infrapatellar fat pad mesenchymal stem cells show immunomodulatory exosomal signatures
Source: Sci Rep. 2022 Mar 4;12:3609. doi: 10.1038/s41598-022-07569-7 (PMC8897449; doi:10.1038/s41598-022-07569-7)

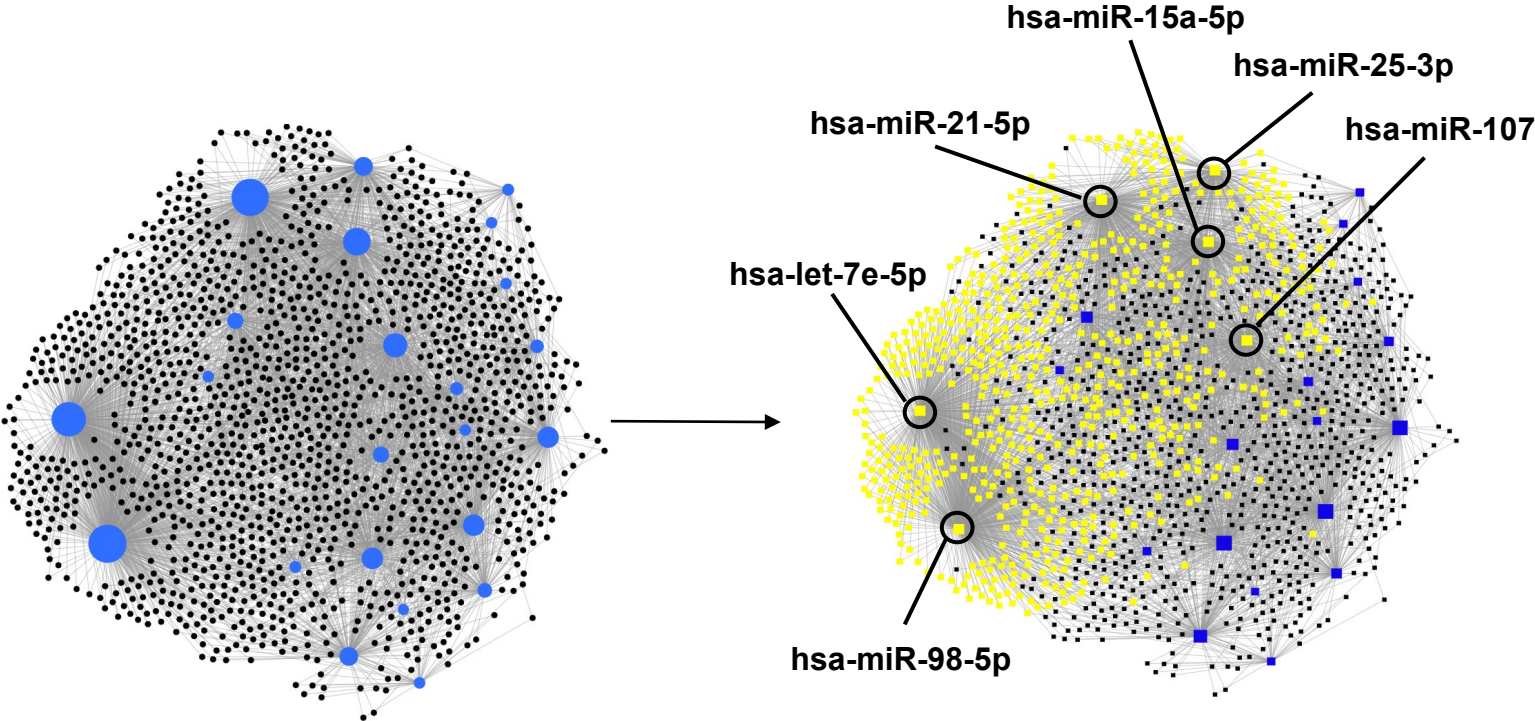

Supplement: Supplementary file 1 — Supplementary Figure S1. [file 41598_2022_7569_MOESM1_ESM.pdf]
