# Supplementary material for: Human infrapatellar fat pad mesenchymal stem cells show immunomodulatory exosomal signatures
Source: Sci Rep. 2022 Mar 4;12:3609. doi: 10.1038/s41598-022-07569-7 (PMC8897449; doi:10.1038/s41598-022-07569-7)
Supplement: Supplementary file 2 — Supplementary Legends. [file 41598_2022_7569_MOESM2_ESM.docx]

**Supplementary Figure 1.** miRNA interactome analysis revealed 6 miRNAs with higher node degree that act as hubs in a gene network.
